# Zero Order Spectrophotometric Method for Estimation of Escitalopram Oxalate in Tablet Formulations

**DOI:** 10.4103/0975-1483.71626

**Published:** 2010

**Authors:** S Sharma, H Rajpurohit, C Sonwal, A Bhandari, VR Choudhary, T Jain

**Affiliations:** *Faculty of Pharmaceutical Sciences, Jodhpur National University, Jodhpur - 342 008, India*; 1 *Rajasthan Drug and Pharmaceutical Ltd, V.K.I. A. Road no. 12, Jaipur - 302 013, India*

**Keywords:** Escitalopram oxalate, validation, zero order spectrophotometry

## Abstract

A new, simple, fast and reliable zero order spectrophotometric method has been developed for determination of Escitalopram Oxalate in bulk and tablet dosage forms. The quantitative determination of drug was carried out using the zero order values (absorbance) measured at 238 nm. Calibration graph constructed at 238 nm was linear in concentration range of 2-20 µg/ml with correlation coefficient 0.9999. The method was found to be precise, accurate, specific, and validated as per ICH guidelines and can be used for determination of Escitalopram Oxalate in tablet formulations.

## INTRODUCTION

Escitalopram Oxalate, chemically, S(+)-1-(3-dimethylaminopropyl)-1-(4-fluorophenyl)-1, 3-dihydroisobenzofuran-5-carbonitrile hydrogen oxalate, is the pure S-enantiomer (single enantiomer) racemic bicyclic phthalane derivative of citalopram used as an antidepressant. The molecular weight of Escitalopram Oxalate is 414.43. Escitalopram Oxalate is white crystalline powder having melting point 146-149°C. It is Soluble in alcohol, sparingly soluble in water and slightly soluble in acetone. The antidepressant and antiobsessive-compulsive actions of escitalopram are presumed to be linked to its inhibition of CNS neuronal uptake of serotonin. Escitalopram blocks the reuptake of serotonin at the serotonin reuptake pump of the neuronal membrane, enhancing the actions of serotonin on 5HT_1A_ auto receptors.[1,2] Escitalopram oxalate is not official in any pharmacopoeia; hence no official method is available for estimation of Escitalopram oxalate in tablet formulation. Only chiral liquid chromatography and high-performance thin-layer chromatographic methods have been developed for determination of Escitalopram Oxalate individually.[3,4]

Moreover, the literature survey revealed that, no method has been reported for estimation of Escitalopram Oxalate in dosage forms by spectrophotometric method individually so far; hence an attempt has been made to develop a simple, accurate, and economic analytical method. The papers describe zero order spectrophotometric method for the estimation of Escitalopram Oxalate in tablet, using 80% (v/v) aqueous methanol as solvent.

## MATERIALS AND METHODS

### Materials

#### Instrument

A UV-VIS Spectrophotometer, model Shimadzu 1601 was employed with spectral bandwidth of 1.8 nm and a wavelength accuracy of ±0.5 nm with a pair of matched quartz cells of 10mm optical path length. Digital weighing balance model HR 200 (Afcoset) and ultra sonic bath, SW 45 (Toshcon/ Tosniwal) was also used.

Escitalopram oxalate was obtained as a gift sample from DD Pharmaceuticals. Sitapura, Jaipur (Raj.). Methanol was procured AR grade, (Merck) and Distilled water (In house production) was used for preparing solutions. 80% v/v methanol in water was selected as a reference solvent. All other reagents and chemicals were of analytical reagent grade.

### Methods

#### Selection and optimization of solvent system

10 mg Escitalopram Oxalate was dissolved in solvent system of different ratio of methanol and water (2:8, 6:4 and 8:2) and final volume was adjusted to 100 ml and allowed to stand for 4-5 hours. Each sample was visually examined and scanned under double beam UV-visible spectrophotometer. The solvent system having methanol and water (8:2) was selected because drug gave optimum absorbance value with better stability and a clear transparent solution was obtained in this solvent system.

#### Preparation of standard solution

**Figure 1:**
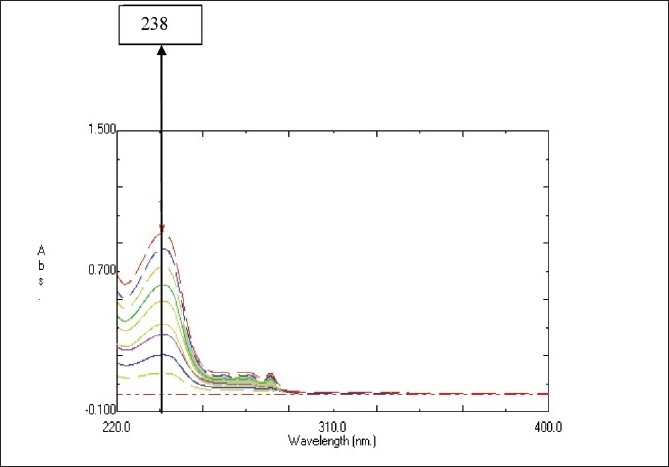
Overlain spectra of 2, 4, 6, 8, 10, 12, 14, 16, and 18 µg/ml of Standard Escitalopram Oxalate Solutions in 80%v/v aqueous methanol

Standard stock solution containing Escitalopram Oxalate was prepared by dissolving 10 mg of Escitalopram Oxalate in 100 ml of reference solvent to obtain stock solution containing 100 µg/ml of drug. Standard stock solution was diluted with reference solvent in a series to obtain working standard solutions in concentration range of 2-20 µg/ml. Solutions were scanned in the UV range [Figure 1] and calibration curve was plotted between absorbance and concentrations.

#### Preparation of sample solution:

Ten tablets each of label claimed 20 mg of Escitalopram Oxalate (Brand I) and label claimed 10 mg of Escitalopram Oxalate (Brand II) were triturated into fine powder. Tablet powder equivalent to 20 mg (Brand I) and 10 mg (Brand II) of Escitalopram Oxalate was transferred to 100 ml volumetric flask separately and dissolved in reference solvent by sonicating for 20 min. The final volume was adjusted up to the mark with reference solvent to give stock solution of 200 µg/ml (Brand I) and 100 µg/ml (Brand II). These solutions were filtered through whatmann filter paper and further diluted suitably to obtain six replicates of 20 µg/ml solutions (Brand I) and 10 µg/ml solutions (Brand II). These solutions were analyzed using spectrophotometer.

## VALIDATION OF PROPOSED METHOD[5–8]

### Specificity

For specificity study, any possible bias was determined by comparison of result of assay value obtained in presence of excipients to assay value without excipients mixture. Assay bias was evaluated by calculating the percentage interference.

### Linearity study

Linearity of the method was investigated by diluting the stock solutions to give a concentration range of 2-20µg/ml. The calibration curve was constructed at optimum experimental conditions by plotting absorbance against concentration.

## REPEATABILITY

Repeatability is performed by intra and inter - day precision. Intra-day precision was determined by analyzing the three different concentrations of drug for three times in the same day. Inter-day precision was determined by analyzing the three different concentrations of the drug for three days in a week.

## RECOVERY STUDY

To study validity and reproducibility of proposed method, recovery studies were carried out by adding known amount of drug to preanalysed sample at three different levels (80%, 100% and 120%) and the percentage mean recoveries of Escitalopram Oxalate in Brand I and Brand II were calculated.

### Limits of Detection (LOD) and Limit of Quantification (LOQ)

The LOD and LOQ were separately determined based on the standard calibration curve. To estimate the LOD and LOQ, diluent was scanned under UV region six times and the signal-to-noise ratio (S/N) was determined. LOD and LOQ were regarded as the amounts for which S/N was 3:1 and 10:1, respectively.

## RESULTS AND DISCUSSION

The zero order spectra of Escitalopram Oxalate were recorded between 200 and 400 nm and the maximum wavelength of Escitalopram Oxalate in 80%v/v aqueous methanol was found to be 238 nm. A clear transparent solution and stable spectra of Escitalopram Oxalate was obtained in methanol: water (8:2) for more than 240 minutes showing stability of Escitalopram Oxalate in this solvent system. Escitalopram Oxalate follows linearity in the concentration range of 2-20 µg/ml with the regression equation y=0.0488×-0.0006, where y is amplitude of the peak at 238 nm of zero order spectra and × is the concentration of the sample in µg/ml. High value of correlation coefficient (0.9999) indicates good linearity and adherence of method to Beer’s law within the concentration range tested by this method [Table 1]. The molar absorptivity of Escitalopram Oxalate was found 20.222×103 Lit/mole/cm. All parameters of the proposed method were validated as per the ICH guidelines. Average interference and relative standard deviation was found to be 0.237% and 0.069%, respectively. Specificity studies were good with results, indicating that the excipients did not interfere with the analysis.

**Table 1:** Beer’s law data and regression characteristics of escitalopram oxalate

Parameters	UV spectrophotometric methods
Regression equation	Y=0.0488×-0.0006
Slope (*b*)	48.8×10^-3^
Intercept (*a*)	0.6×10^-3^
Linearity range (µg /ml)	2.003-20.032
Molar absorptivity (Lit/mole/cm)	20.222×10^3^
Correlation Coefficient (r)	0.9999

The recovery of drug was determined at 80, 100 and 120 % level. The percent mean recovery was obtained 98.99 for Brand I and 99.12 for Brand II. Percent mean recoveries greater than 99% with low standard deviation justifies the accuracy of the method. The percent mean recovery lies within the desirable confidence interval of 98-102%, hence it can be said that the proposed method is accurate. The calibration curve was repeated three times in a day at three different concentrations and the calibration curve was repeated three times on three different days at three different concentrations. These values confirmed the intra-day and inter-day precision of the method. The intra-day and inter-day % RSD values were calculated which were found to be in the range of 0.176-0.177.[Table 2] The low %RSD value revealed that the proposed method at selected wavelength is precise. The calculated limit of detection (LOD) and limit of quantification (LOQ) were found to be 0.160 (µg/ml) and 0.534 (µg/ml) respectively. The LOD and LOQ showed that the method is sensitive for Escitalopram Oxalate.

**Table 2:** Summary of validation parameters for estimation of escitalopram oxalate by UV-spectroscopy

Parameter	Observation
	Escitalopram oxalate
Specificity	No interference was found w.r.t.# excipients
Linearity (Correlation coefficient r)	0.9999
Accuracy* (% Recovery)	98.99% and 99.95%
Precision RSD%	
Repeatability (n= 6)	0.803
Intra-day (n=3)	0.176
Inter-day (days=3)	0.177
LOD (µg/ml)	0.160
LOQ (µg/ml)	0.534

* Average of three determinations,

# With Respect To

Two brands of tablets were analyzed and amount of drugs was determined by proposed method; it was in good agreement with the label claim. [Table 3] The proposed spectrophotometric method for estimation of Escitalopram oxalate was found to be simple, precise, accurate and sensitive and can be utilized as a quality control tool for estimation of Escitalopram Oxalate in tablet dosage form.

**Table 3:** Assay of Marketed Formulation

Brand	Labeled amount (mg)	Amount found* (mg)	% of Labeled amount*	%RSD
ESTA 20	20	19.620.084	98.13±0.257	0.429
S CITADEP-10	10	9.93±0.025	99.17±0.421	0.259

* Average of six estimations
